# Interfacial Regulation of Hard-Anodized Aluminum Alloy for Fabrication of a Wear-Resistant Superhydrophobic F-SiO_2_@PU Composite Coating with Enhanced Long-Term Corrosion Protection

**DOI:** 10.3390/ma19194155

**Published:** 2026-09-29

**Authors:** Hong-Bo Wang, Miao Yu, Chong Ren, Muhaiminul Islam Shakil, Chen Chen, Liang Zhou, Da-Yan Ma, Wei Wang

**Affiliations:** 1 School of Materials Science and Engineering, Chang’an University, Xi’an 710064, China; 2 Shaanxi Key Laboratory of Carbon Dioxide Sequestration and Enhanced Oil Recovery, Xi’an 710075, China; 3 State Key Laboratory for Mechanical Behavior of Materials, Xi’an Jiaotong University, Xi’an 710049, China; 4 State Key Laboratory of Clean and Efficient Turbomachinery Power Equipment, Dongfang Turbine Co., Ltd., Deyang 618000, China

**Keywords:** aluminum alloy, superhydrophobic coating, wear resistance, corrosion protection

## Abstract

Conventional superhydrophobic coatings on aluminum alloys are frequently limited by inadequate coating–substrate adhesion, poor mechanical robustness, and the vulnerability of their surface micro/nanostructures to mechanical damage. To address these challenges, a hydroxyl-rich anodic oxide interlayer was formed via hard anodization followed by a hydroxylation treatment, thereby enhancing interfacial bonding and providing abundant active anchoring sites for coating deposition. Using this interface-engineering strategy, a mechanically durable superhydrophobic F-SiO_2_@polyurethane (PU) composite coating with hierarchical micro/nanostructures was successfully fabricated on aluminum alloy substrates through a facile spray-coating process. The anodized substrate provides abundant hydroxyl groups and interfacial anchoring sites, while the PU matrix enhances structural integrity and facilitates particle fixation. Incorporating fluorinated SiO_2_ nanoparticles produced a hierarchical micro/nanostructure with low surface energy. After 10,000 cm of abrasion and 200 tape-peeling cycles, the coating maintained contact angles above 150°. The coating maintained superior corrosion resistance during prolonged immersion in acidic, alkaline, and saline environments. The improved durability and corrosion resistance originate from the synergistic effects of strong interfacial bonding, polymer barrier protection, and the fluorinated hierarchical structure. This study offers a promising strategy for developing durable superhydrophobic coatings for long-term protection of aluminum alloys.

## 1. Introduction

Aluminum alloys are among the most extensively utilized lightweight structural materials owing to their high specific strength, excellent thermal and electrical conductivity, and superior machinability [1,2,3]. These attributes have promoted their widespread application in aerospace engineering, marine equipment, transportation systems, and electronic packaging [4,5]. Nevertheless, the native oxide film on aluminum alloys is relatively thin and vulnerable to mechanical damage, chloride-ion penetration, and chemical attack [6,7]. Under aggressive service conditions, such as high humidity, saline atmospheres, acidic or alkaline media, and cyclic environmental fluctuations, localized degradation processes, including pitting corrosion, crevice corrosion, and intergranular corrosion, can be readily initiated [6,7,8]. Once the passive film is disrupted, corrosion propagates rapidly through electrochemical reactions, resulting in material loss, deterioration of mechanical integrity, and eventual structural failure [9]. Corrosion-induced damage not only shortens the service life of aluminum alloy components but also compromises the operational safety and reliability of engineering systems [10]. Therefore, effective surface protection strategies with long-term corrosion resistance and environmental adaptability are urgently needed for the practical application of aluminum alloys.

Inspired by the micro/nanostructures and low surface energy of natural biological interfaces such as lotus leaves [11], dandelion seeds [12], water striders [13], and springtails [14], superhydrophobic surface engineering provides a new design concept for the protection of aluminum alloys. By constructing micro/nanoscale rough structures and combining them with low-surface-energy modification, a stable gas film can be formed on the surface, effectively blocking the contact between the substrate and corrosive media, thereby achieving self-cleaning and anti-corrosion functions [15,16,17].

To address these issues, research worldwide mainly focuses on enhancing the mechanical properties of the coating and optimizing the organic-inorganic composite interface [18,19,20]. One approach enhances the coating hardness and scratch resistance by incorporating inorganic hard particles such as SiO_2_ and Al_2_O_3_ [18,21]. However, this strategy often increases coating brittleness and compromises deformation coordination, making the coating prone to microcracks and localized failure under bending or impact loads [22]. Another approach involves directly introducing inorganic particles into the polymer matrix, enhancing their dispersion and interfacial bonding through surface modification [23]. However, during the modification process, the consumption of surface-active groups weakens the chemical bonding between the particles and the polymer, making the interfacial bonding more dependent on weak physical forces. Under friction and shear stress, the particles are prone to detachment and form interfacial defects, accelerating the penetration of water and ions [19,23]. From an essential perspective, the core bottleneck of the existing system lies in the lack of a multi-scale, continuous, and stable interface chemical coupling mechanism between the organic matrix, inorganic fillers, and metal substrate [24].

Among various polymeric matrices, PU has attracted extensive attention in the field of protective coatings owing to its excellent flexibility, strong adhesion, outstanding mechanical toughness, and good environmental stability [25,26]. The presence of abundant urethane linkages and reactive functional groups enables PU to form strong interfacial interactions with both inorganic fillers and metallic substrates. Moreover, its elastomeric network can effectively dissipate external stress through elastic deformation, thereby alleviating stress concentration and suppressing crack initiation and propagation during mechanical loading [27]. These characteristics make PU a promising candidate for constructing durable superhydrophobic coatings with enhanced wear resistance and corrosion protection [28]. Nevertheless, the intrinsic surface energy of PU remains relatively high, making it difficult to achieve stable superhydrophobicity without the incorporation of micro/nanoscale rough structures and low-surface-energy components [29]. Furthermore, the interfacial compatibility between modified inorganic particles and the PU matrix is often insufficient, and the bonding strength at the coating–substrate interface remains limited [30]. As a result, particle detachment, interfacial debonding, and structural degradation may still occur under long-term mechanical wear and corrosive environments, leading to a gradual deterioration of protective performance.

To address the challenges of insufficient interfacial stability and the difficulty in simultaneously achieving high wear resistance and long-term corrosion protection, a hierarchical F-SiO_2_@PU composite coating was developed on 6061 aluminum alloy by integrating a hard-anodized substrate, a particle-reinforced PU interlayer, and a fluorinated F-SiO_2_ top layer. An anodic oxide layer with a porous architecture and abundant hydroxyl groups was first constructed to regulate surface reactivity and enhance interfacial bonding. Subsequently, FDTS-modified SiO_2_ nanoparticles were incorporated into the PU matrix to establish a stable hierarchical micro/nanostructure with low surface energy. The hydroxyl-rich anodized surface further reacted with isocyanate groups in the PU matrix, forming a strongly coupled substrate–polymer–particle interface. The resulting coating exhibited excellent mechanical durability, maintaining a water contact angle of 150 ± 2° after 10,000 cm of abrasion and 200 tape-peeling cycles. Moreover, superior corrosion resistance was achieved in 3.5 wt.% NaCl solution, with a low-frequency impedance modulus of log|Z|_0.1 Hz_ = 8.0 and a corrosion current density as low as 5.36 × 10^−11^ A cm^−2^. The enhanced performance arises from the synergistic effects of interfacial stabilization, PU-mediated particle fixation and stress dissipation, and the low-surface-energy air-trapping effect of the fluorinated hierarchical structure. This work provides an effective interfacial regulation strategy for the development of mechanically robust and long-lasting superhydrophobic coatings on aluminum alloys.

## 2. Experimental Section

### 2.1. Materials

In this study, 6061 aluminum alloy plates (20 mm × 20 mm × 2 mm) were employed as substrates. The diatomite particles were purchased from Sainke Experimental Equipment (Jinan) Co., Ltd. (Jinan, China). Aluminum hydroxide and sodium hydroxide were both purchased from Fuchen (Tianjin) Chemical Reagent Co., Ltd. (Tianjin, China), and 1H, 1H, 2H, 2H-perfluorooctyl trichlorosilane (FDTS), and Capstone FS-3100 were all purchased from Aladdin (Shanghai) Reagent Co., Ltd. (Shanghai, China). Tetraethyl orthosilicate (TEOS) was purchased from Tianjin Damao Chemical Reagent Factory. Anhydrous ethanol, deionized water, and aqueous ammonia (25 wt%) were purchased from Tianjin Fuyu Fine Chemical Co., Ltd. (Tianjin, China). Nanosilica sol (30 wt%) was purchased from Guangzhou Fuer Chemical Technology Co., Ltd. (Guangzhou, China). Waterborne polyurethane resin (50 wt%) was purchased from Jining Huakai Resin Co., Ltd. (Jining, China). Hydrochloric acid was purchased from Shenyang Shenyi Reagent Factory (Shenyang, China). Sodium chloride was purchased from Tianjin Yongda Chemical Reagent Co., Ltd. (Tianjin, China).

### 2.2. The Preparation of the Hydroxylated Substrate

Aluminum alloy specimens were mechanically polished and successively subjected to degreasing, alkaline etching, acid neutralization, and desmutting prior to anodization. Hard anodization was performed in a 130 g L^−1^ H_2_SO_4_ electrolyte at 0 °C for 50 min, resulting in the formation of anodic aluminum oxide (AAO) films. The obtained AAO samples were subsequently treated in boiling deionized water to promote hydration of the oxide layer and generate a hydroxyl-rich surface. The thickness of the anodic oxide films, measured using a coating thickness tester, ranged from 60 to 80 μm.

### 2.3. Preparation of Modified Nanoparticles

#### 2.3.1. Preparation of F-Al(OH)_3_@Siltite Nanoparticles

To prepare fluorinated Al(OH)_3_@diatomite particles, 0.3 g of Al(OH)_3_ and 1.0 g of diatomite were dispersed in a premixed solution containing 20 mL of anhydrous ethanol, 2 mL of deionized water, and 1 mL of aqueous ammonia. The suspension was stirred at 50 °C for 1 h to ensure uniform dispersion. Subsequently, 0.5 mL of 1H,1H,2H,2H-perfluorooctyltrichlorosilane (FDTS) was dissolved in 20 g of n-hexane and ultrasonicated for 10 min. The obtained FDTS solution was added dropwise to the suspension, and the mixture was allowed to react at 50 °C for 24 h. During this process, FDTS molecules were grafted onto the surface hydroxyl groups of Al(OH)_3_ and diatomite via silanization, thereby reducing the surface energy of the particles. Finally, the modified particles were dried, ground, and sieved through a 200-mesh screen to obtain fluorinated Al(OH)_3_@diatomite composite particles (F-Al(OH)_3_@diatomite).

#### 2.3.2. Preparation of F-SiO_2_ Solution

To prepare fluorinated SiO_2_ nanoparticles, 0.5 mL of tetraethyl orthosilicate (TEOS) and 1.0 g of silica sol were dispersed in a premixed solution containing 20 mL of anhydrous ethanol, 2 mL of deionized water, and 1 mL of aqueous ammonia. The mixture was stirred at 50 °C for 1 h to obtain a uniform silica precursor suspension. Subsequently, 0.1–0.5 mL of 1H,1H,2H,2H-perfluorooctyltrichlorosilane (FDTS) was dissolved in 20 g of n-hexane and ultrasonically treated for 20 min. The resulting FDTS solution was added dropwise to the precursor suspension, and the mixture was maintained at 50 °C for 24 h. Through the silanization reaction between FDTS and surface hydroxyl groups on the silica particles, fluorinated SiO_2_ nanoparticles with hierarchical roughness and low surface energy were obtained. The resulting suspension was designated as F-SiO_2_.

### 2.4. Preparation of F-SiO_2_@PU Coating

The F-SiO_2_@PU coating was prepared via a two-step spray deposition process. First, 5 g of polyurethane was mixed with 5 mL of deionized water, and 0.03 g of FS-3100 was added to improve wettability and reduce the surface tension of the suspension. Subsequently, 1.3 g of F-Al(OH)_3_@diatomite particles was introduced and uniformly dispersed under magnetic stirring. The resulting suspension was sprayed onto the hydroxylated aluminum substrate to form an F-Al (OH)_3_@diatomite-reinforced PU interlayer, in which the micron-sized composite particles were dispersed within the PU matrix to provide structural reinforcement and support for the subsequent superhydrophobic top layer. Before complete curing of the polyurethane matrix, 5 mL of F-SiO_2_ nanodispersion was deposited onto the coating surface via spray coating. The spraying parameters were set to an air pressure of 0.4 MPa, a spray distance of 15 cm, and three spraying cycles of 5 s each, with 20 s intervals between cycles. This sequential deposition strategy enabled the formation of a robust hierarchical micro/nanostructured surface with low surface energy. The final coating exhibited an average thickness of 120.5 ± 1.2 μm. For simplicity, the composite coating consisting of an F-Al (OH)_3_@diatomite-reinforced PU interlayer and an F-SiO_2_ top layer is hereafter denoted as F-SiO_2_@PU.

### 2.5. Characterization (Instruments and Characterization)

The wettability of the coatings was evaluated by measuring the water contact angle (CA) and sliding angle (SA) using a JC2000D1 contact angle analyzer (Shanghai Zhongchen Digital Technology Equipment Co., Ltd., Shanghai, China). Surface morphology and roughness were characterized by FE-SEM (S-4800, Hitachi, Tokyo, Japan) and white-light interferometry, respectively. The chemical composition, surface functional groups, and thermal stability of the coatings were analyzed by XPS, FTIR, and thermogravimetric analysis (TGA). Corrosion resistance was assessed through immersion tests in 0.1 M HCl, 1 M NaOH, and 3.5 wt.% NaCl solutions, together with potentiodynamic polarization and electrochemical impedance spectroscopy (EIS) measurements performed on a VersaSTAT-3F electrochemical workstation (AMETEK, Berwyn, PA, USA). The mechanical durability of the coatings was evaluated using a sandpaper abrasion test with 2000-grit SiC sandpaper under a 200 g load. For each abrasion distance and each peeling cycle, at least three independent coated samples were tested under identical conditions. The CA and SA values are reported as mean ± standard deviation, and the error bars in Section 3.3 represent the standard deviation of three independent measurements. The tape-peeling test was also repeated three times independently to confirm reproducibility.

## 3. Results and Discussion

### 3.1. Morphology and Chemical Composition of Fluorinated SiO_2_ Nanoparticles

**Figure 1:**
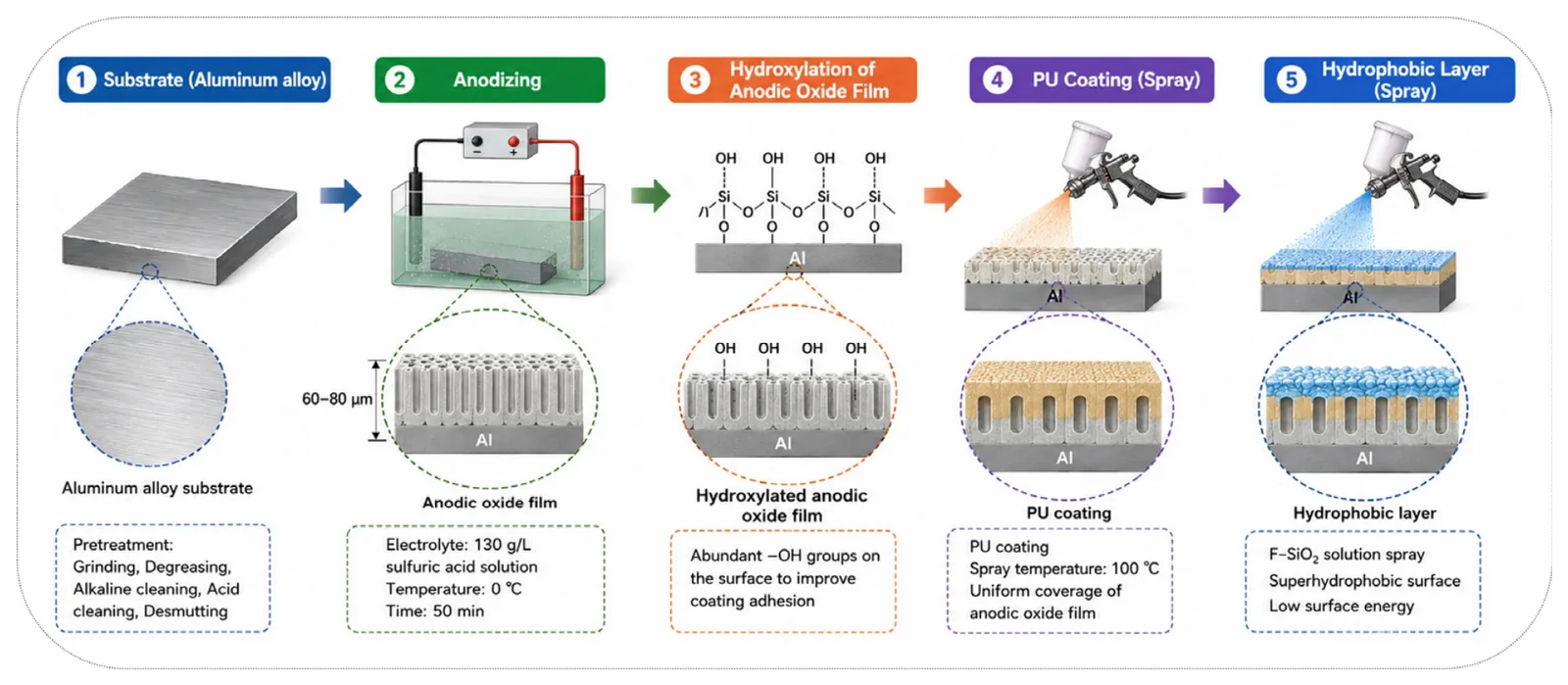
Preparation flowchart of F-SiO_2_@PU composite coating.

As illustrated in Figure 1, the Formation mechanism of the F-SiO_2_@PU superhydrophobic coating on anodized aluminum alloy is as follows: A porous anodic oxide film is first generated on the aluminum alloy surface through anodization, providing a rough micro/nanostructure and abundant anchoring sites. Subsequent hydroxylation treatment generates a hydrated surface containing abundant polar groups, which is favorable for interfacial interactions with the PU matrix. Meanwhile, the rough and porous anodic surface provides additional anchoring sites for coating deposition. The PU matrix forms a continuous barrier layer and facilitates the fixation of functional particles. Finally, fluorinated SiO_2_ nanoparticles are deposited onto the PU surface, constructing a hierarchical micro/nanostructure with low surface energy. The combined effects of interfacial interactions, PU-mediated particle fixation, and trapped air within the hierarchical rough structure effectively suppress electrolyte penetration, thereby improving the mechanical durability and corrosion resistance of the coating.

SiO_2_ tends to agglomerate because of its high surface energy, while FDTS modification can effectively reduce the surface energy and regulate particle morphology. As shown in Figure S1a–c, increasing the FDTS dosage from 0.1 to 0.3 mL gradually transformed the SiO_2_ particles from a loose distribution into a more uniform and well-defined packed structure, accompanied by the development of hierarchical micro/nanoscale roughness. At 0.3 mL FDTS (Figure S1c), the particles exhibited clear boundaries and relatively uniform pores, resulting in a CA of 161.5 ± 1.5° and an SA of 1.5 ± 0.5°. At 0.4 mL FDTS (Figure S1d), particle adhesion became more pronounced, accompanied by partial agglomeration and flocculent structures. At 0.5 mL (Figure S1e), severe secondary agglomeration led to larger block-like and locally compact aggregates, partially obscuring the original particle morphology. This behavior can be attributed to enhanced self-condensation and bridging of excessive silane molecules, which disrupt the uniform hierarchical structure. Therefore, 0.3 mL of FDTS was selected for SiO_2_ modification to achieve optimal superhydrophobic performance.

**Figure 2:**
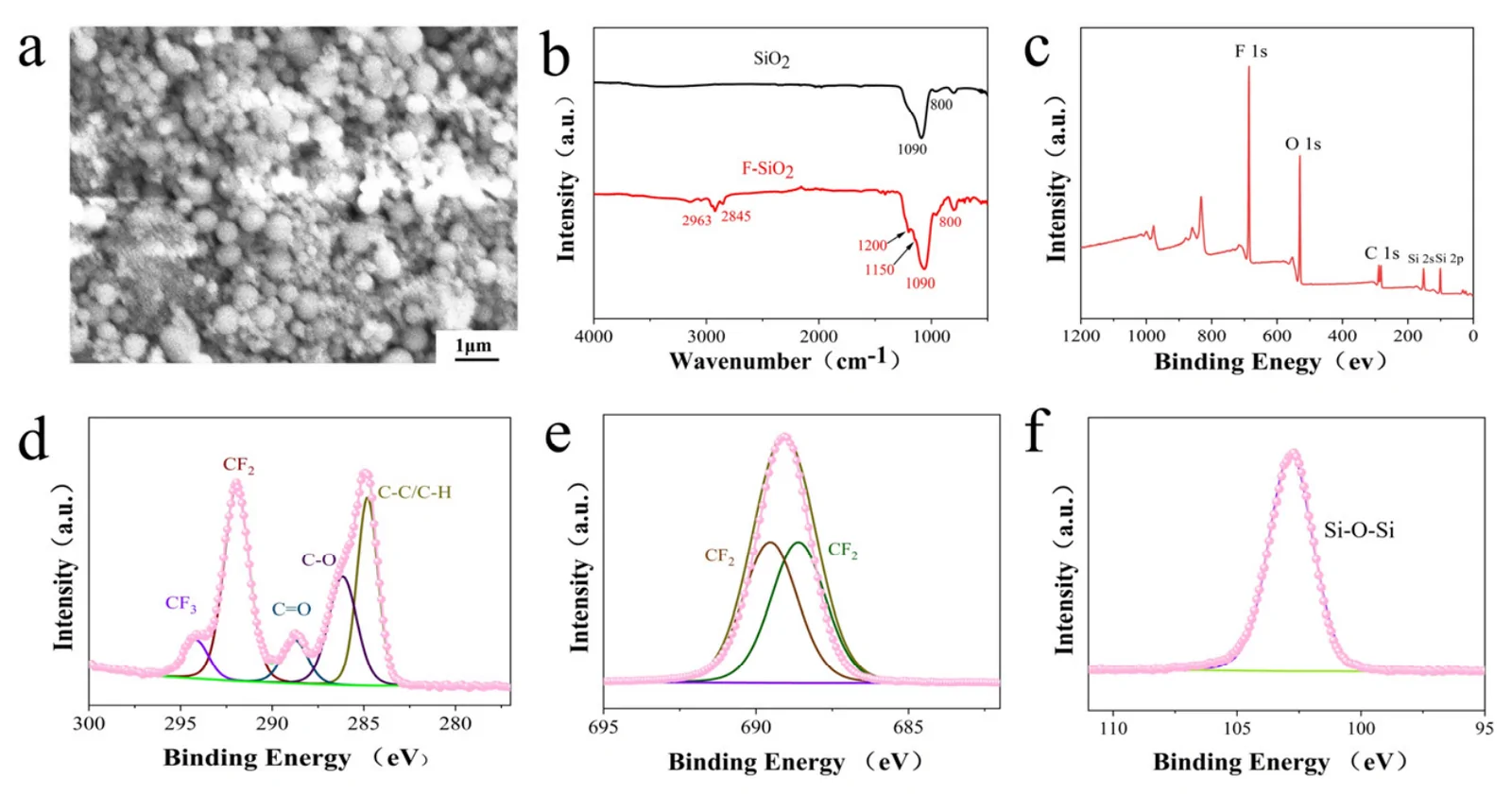
(**a**) The SEM image of F-SiO_2_; (**b**) FTIR spectra of SiO_2_ and F-SiO_2_; (**c**) XPS spectrum of F-SiO_2_; (**d**–**f**) high-resolution spectra of C1s, F1s, and O1s in F-SiO_2_, respectively.

The surface morphology of the SiO_2_ nanoparticles after fluorination is shown in Figure 2a. Following FDTS treatment, the particles maintained their spherical morphology while developing a rougher nanoscale texture due to the formation of a fluorinated silane layer. The chemical composition of the particles was further analyzed using FTIR and XPS. The FTIR spectra of F-SiO_2_ exhibited characteristic absorption bands corresponding to fluorinated alkyl groups, confirming the successful introduction of fluorosilane molecules onto the silica surface. The FTIR spectra of unmodified SiO_2_ and F-SiO_2_ are shown in Figure 2b. The absorption peaks at 1090 cm^−1^ and 800 cm^−1^ for unmodified SiO_2_ correspond to the asymmetric and symmetric stretching vibrations of Si-O-Si bonds [31], respectively. These are characteristic peaks of a silica framework that demonstrate the presence of a complete siloxane network in the prepared particles. After FDTS modification, the samples retained the characteristic Si-O-Si absorption peaks near 1090 cm^−1^ and 800 cm^−1^ while exhibiting new peaks at 2963 cm^−1^ and 2845 cm^−1^, corresponding to the stretching vibrations of the -CH_2_ groups in the organic segments; additionally, peaks at 1200 cm^−1^ and 1150 cm^−1^ represent the stretching vibrations of C-F bonds [32].

XPS analysis further verified the presence of fluorine-containing species through the appearance of distinct F 1s signals. The full XPS spectrum of F-SiO_2_ (Figure 2c) clearly exhibits a series of characteristic electronic transition signals, including the F 1s peak at 689.2 eV, the C 1s peak at 284.8 eV, and the O 1s peak at 533.8 eV, as well as the Si 2s and Si 2p peaks at 153.6 eV and 103.6 eV, respectively. The presence of the highly intense F-related characteristic signal in these spectral lines provides preliminary and compelling qualitative evidence that the FDTS molecule has successfully been introduced and coated onto the surface layer of the nanosilica particles. The high-resolution C 1s energy spectrum is shown in Figure 2d. Sub-peaks at 284.8 eV, 286.3 eV, and 288.5 eV correspond to the C-C (or C-H), C-O, and C=O bonds, respectively. Peaks at 291.6 eV and 293.5 eV in the higher-energy region are attributed to the -CF_2_ groups and terminal -CF_3_ groups within the fluorinated organic chain [33]. Due to fluorine’s strong electronegativity, the electron cloud density around the connected carbon atoms decreases significantly, resulting in a substantial shift of their core electron binding energies toward higher values. The high-resolution F1s spectrum is shown in Figure 2e, where the characteristic peaks at -CF_2_- (689.6 eV) and -CF_3_- (688.9 eV) indicate that the fluorinated segments in the FDTS molecule were preserved during modification, and the main peak at O1s (Figure 2f) primarily corresponds to Si-O-Si (533.8 eV), demonstrating that the modified sample retains a stable silica framework. The increased carbon and fluorine content observed after modification provided direct evidence for the covalent grafting of FDTS onto the SiO_2_ surface.

**Figure 3:**
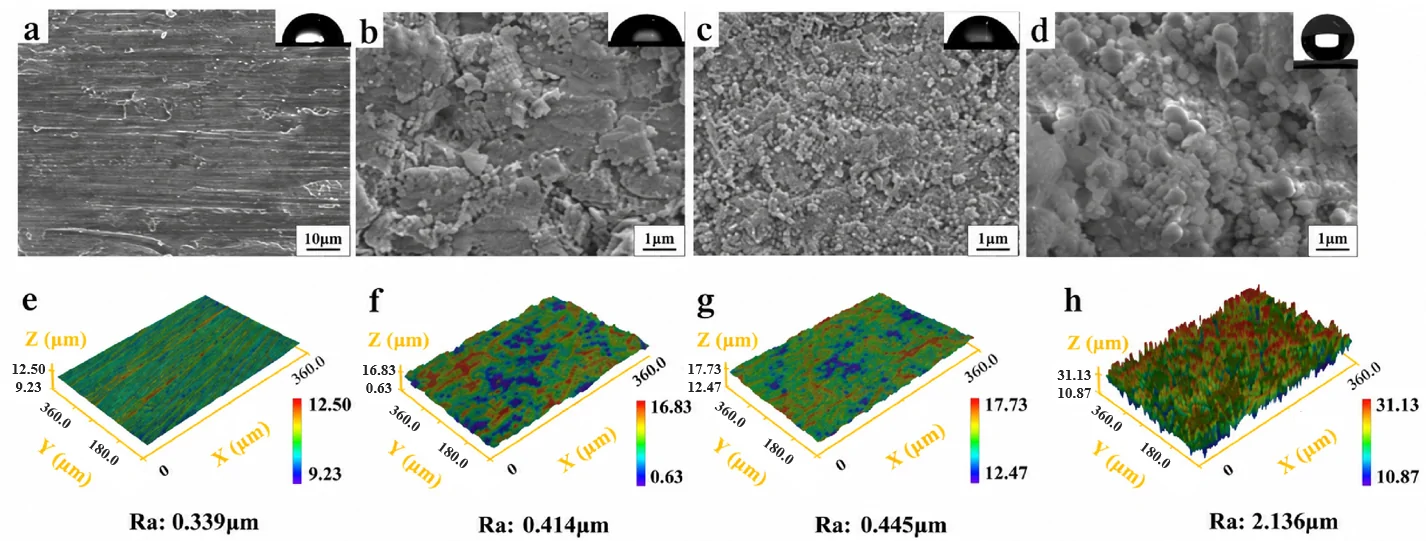
SEM images of (**a**) the Al substrate, (**b**) anodized sample, (**c**) hydroxylated sample, and (**d**) F-SiO_2_@PU sample; (**e**–**h**) white-light interference images of aluminum alloy, anodized sample, hydroxylated sample, and F-SiO_2_@PU-coated sample, respectively.

Figure 3 illustrates the surface morphology of aluminum alloy substrates after anodizing, hydroxylation, and F-SiO_2_@PU coating treatment. Figure 3a shows the aluminum alloy substrate with mechanical scratches on its surface, exhibiting a contact angle of 95°. The anodized surface (Figure 3b) exhibits a rough, porous structure composed of irregular oxide flakes and particles. After hydroxylation (Figure 3c), numerous fine hydration products form on the surface, partially filling the pores and resulting in a denser, more uniform morphology. This change is attributed to the hydration of Al_2_O_3_ into boehmite, thereby enhancing the density of the oxide layer. Upon deposition of the F-SiO_2_@PU coating (Figure 3d), the uniform distribution of SiO_2_ nanoparticles within the polyurethane matrix creates a hierarchical micro/nanostructure. Increased surface roughness facilitates air trapping at the solid–liquid interface, promoting the formation of a stable Cassie–Baxter state; this leads to a contact angle of 161.5° and improved superhydrophobicity and corrosion resistance of the coating. To further clarify the role of PU in the composite structure, the surface morphologies of the F-SiO_2_ and F-SiO_2_@PU coatings were comparatively examined (Figure S2). Compared with the loosely packed F-SiO_2_ coating, the F-SiO_2_@PU coating exhibited a more continuous and compact morphology, with some nanoparticles embedded in or encapsulated by the PU matrix. This observation indicates that the PU network effectively immobilizes the functional particles and improves the structural integrity of the composite coating.

Figure 3e–h demonstrate white-light interferometric morphology images and surface roughness measurements of aluminum alloy substrates, anodized samples, hydroxylated samples, and the F-SiO_2_@PU composite superhydrophobic coatings, respectively. These images reveal significant variations in three-dimensional surface characteristics among samples under different conditions, with surface roughness increasing progressively. The aluminum alloy substrate exhibits a relatively smooth surface with minimal ripples; distinct parallel striations are observed along specific directions, accompanied by slight peak-valley variations, without pronounced protrusions or depressions, with an average surface roughness (Ra) of only 0.339 μm. After anodization (Figure 3f), the original directional texture diminishes, giving way to a more uniform planar relief structure with slightly increased roughness, indicating that anodization establishes an initial framework of roughness on the substrate surface. Hydroxylation treatment further modifies the surface morphology: while hydration products (boehmite) fill deep pores, they create densely distributed particulate protrusions, resulting in a dense interlocking peak–valley pattern with Ra rising to 0.445 μm—demonstrating that pore sealing enhances surface roughness. The incorporation of F-SiO_2_@PU introduces additional complexity: instead of simple continuous ripples, the surface displays densely arranged micro-protrusions and interconnected peak–valley structures, forming a more intricate three-dimensional profile with significantly elevated surface roughness (Ra = 2.135 μm).

**Figure 4:**
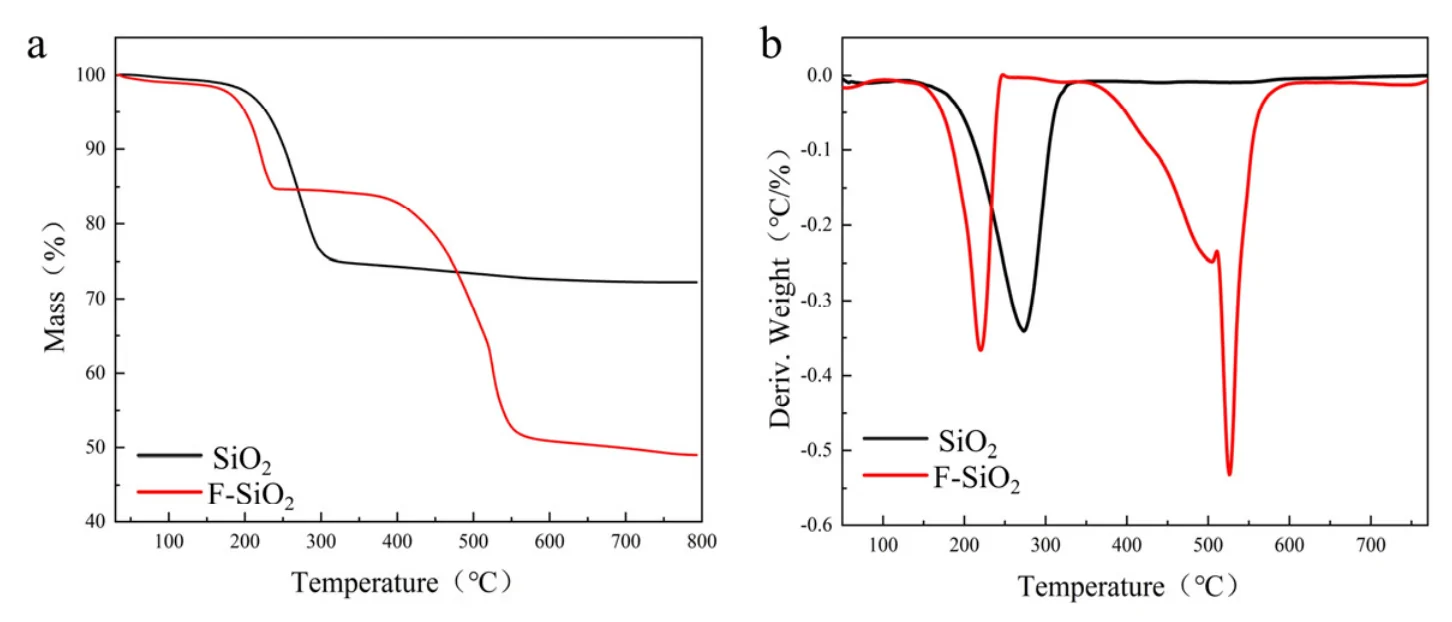
(**a**) TGA curves and (**b**) DTG curves of SiO_2_ and F-SiO_2_.

Thermogravimetric analysis was conducted to evaluate the thermal stability of the modified nanoparticles. As shown in Figure 4a, pristine SiO_2_ exhibited a relatively lower total weight loss of approximately 28% from 100% to 72% over the range of 50–800 °C. The main weight-loss stage started at approximately 200 °C and ended at approximately 300 °C, with a mass loss of approximately 22%, which is associated with the condensation of surface hydroxyl groups and the removal of residual adsorbed species. In contrast, F-SiO_2_ displayed a distinct two-step decomposition behavior with a total weight loss of approximately 52% (from 100% to 48%). The first weight-loss stage started at approximately 150 °C and ended at approximately 250 °C, with a mass loss of approximately 14%, mainly attributed to the evaporation of residual solvents and low-molecular-weight siloxane species [34]. After this stage, only a slight weight loss of about 2% occurred between 250 and 400 °C, indicating good thermal stability of the residual silica framework. The second weight-loss stage started at approximately 400 °C and ended at approximately 550 °C, with a mass loss of approximately 32%, corresponding to the decomposition of the grafted fluorinated alkyl chains from FDTS [35]. The total weight loss of F-SiO_2_ was about 24% higher than that of pristine SiO_2_, indicating the successful grafting of FDTS onto the SiO_2_ surface. Above 550 °C, only a slight further weight loss of about 3% was observed.

Collectively, the morphology, chemical composition, and thermal analysis results confirm that FDTS molecules were successfully grafted onto the SiO_2_ nanoparticles. The resulting fluorinated particles possess both low surface energy and enhanced nanoscale roughness, providing the fundamental building blocks required for constructing durable superhydrophobic coatings.

### 3.2. Wettability and Self-Cleaning Performance of F-SiO_2_@PU Composite Coating

**Figure 5:**
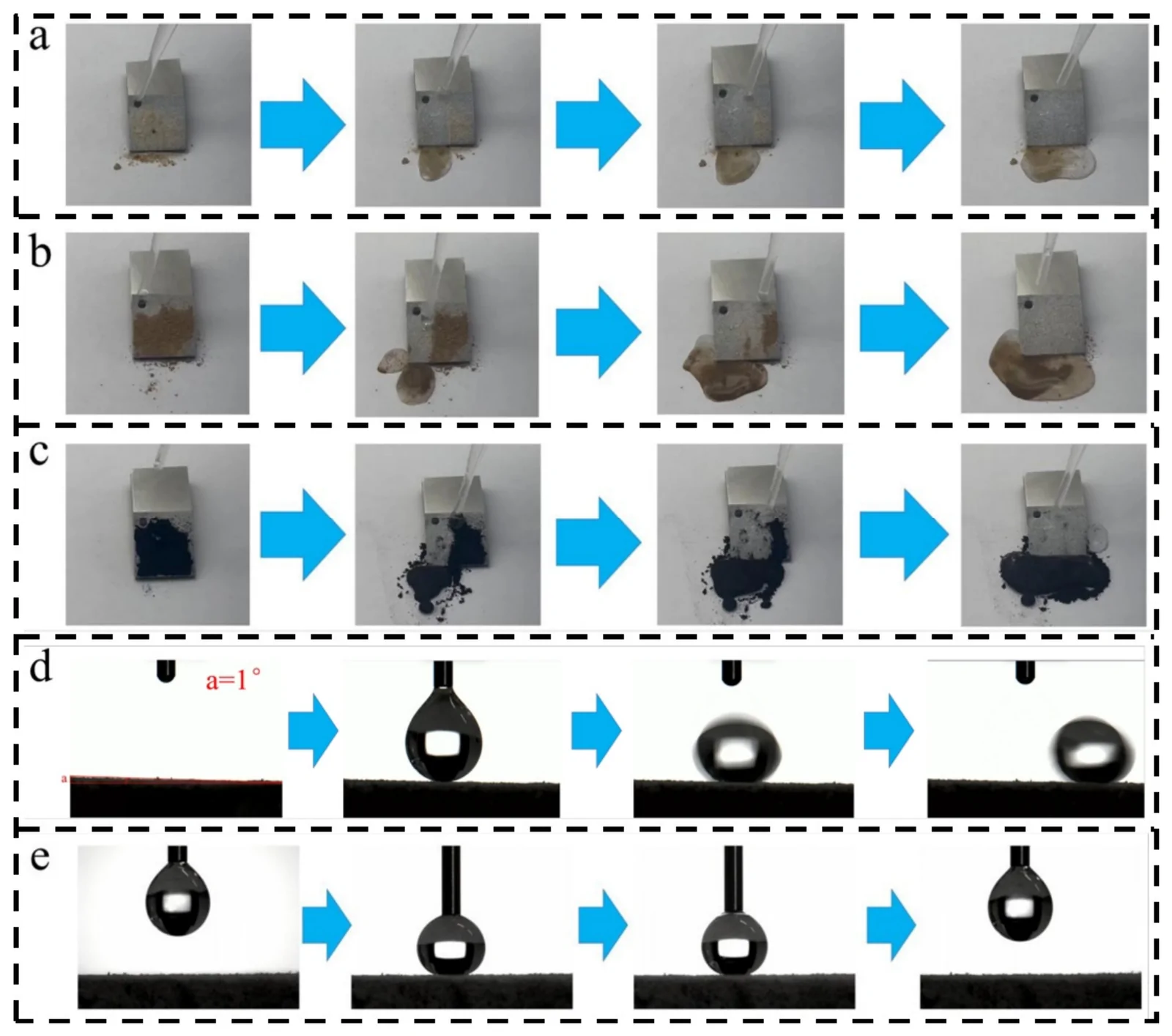
Self-cleaning images of the F-SiO_2_@PU coating: (**a**) sand, (**b**) soil, (**c**) carbon black, (**d**) rolling angle, and (**e**) water droplet non-adhesion test.

The wettability and self-cleaning behavior of the F-SiO_2_@PU composite coating were systematically investigated to evaluate its surface functionality. Representative self-cleaning tests using sand, soil, and carbon black as contaminants are shown in Figure 5. Regardless of the contaminant type, water droplets readily rolled across the coating surface and effectively removed adhered particles, leaving the surface clean. This phenomenon demonstrates excellent self-cleaning capability and confirms the successful establishment of a lotus-leaf-inspired superhydrophobic interface [36].

The outstanding self-cleaning performance originates from the hierarchical micro/nanostructure combined with the fluorinated low-surface-energy chemistry. When contaminants are deposited on the coating surface, the actual contact area between the particles and the substrate remains extremely small due to trapped air pockets. As water droplets roll across the surface, capillary forces at the liquid–particle interface exceed the weak adhesion forces between contaminants and the coating, thereby enabling efficient contaminant removal.

The dynamic wetting behavior was further characterized through sliding-angle measurements. As illustrated in Figure 5d, the coating exhibited an ultralow sliding angle of approximately 1°, indicating negligible contact-angle hysteresis and excellent droplet mobility. Such behavior is characteristic of a stable Cassie–Baxter wetting state, in which water droplets rest predominantly on an air-solid composite interface rather than directly contacting the solid substrate.

To further assess liquid–solid adhesion, a droplet adhesion test was performed by compressing and subsequently withdrawing a 5 μL water droplet from the coating surface (Figure 5e). Throughout the loading-unloading process, the droplet maintained a nearly spherical morphology and detached instantly once the external force was withdrawn. No liquid-bridge formation, droplet fragmentation, or residual liquid traces were observed. These observations indicate extremely weak interfacial adhesion and confirm the presence of a stable trapped-air layer at the coating surface.

The superior wetting behavior can be attributed to the synergistic effect of the fluorinated SiO_2_ nanoparticles and the PU-supported hierarchical architecture. The fluorinated alkyl chains substantially reduce the surface free energy, whereas the multiscale rough structure amplifies hydrophobicity by stabilizing the Cassie–Baxter state. Simultaneously, the PU matrix enhances particle fixation and structural integrity, thereby preserving the micro/nanostructure required for sustained superhydrophobic performance.

Therefore, the F-SiO_2_@PU composite coating exhibits excellent water repellency, ultralow liquid adhesion, and efficient self-cleaning capability, which are highly desirable characteristics for long-term anti-corrosion and anti-fouling applications.

### 3.3. Mechanical Durability and Environmental Stability

**Figure 6:**
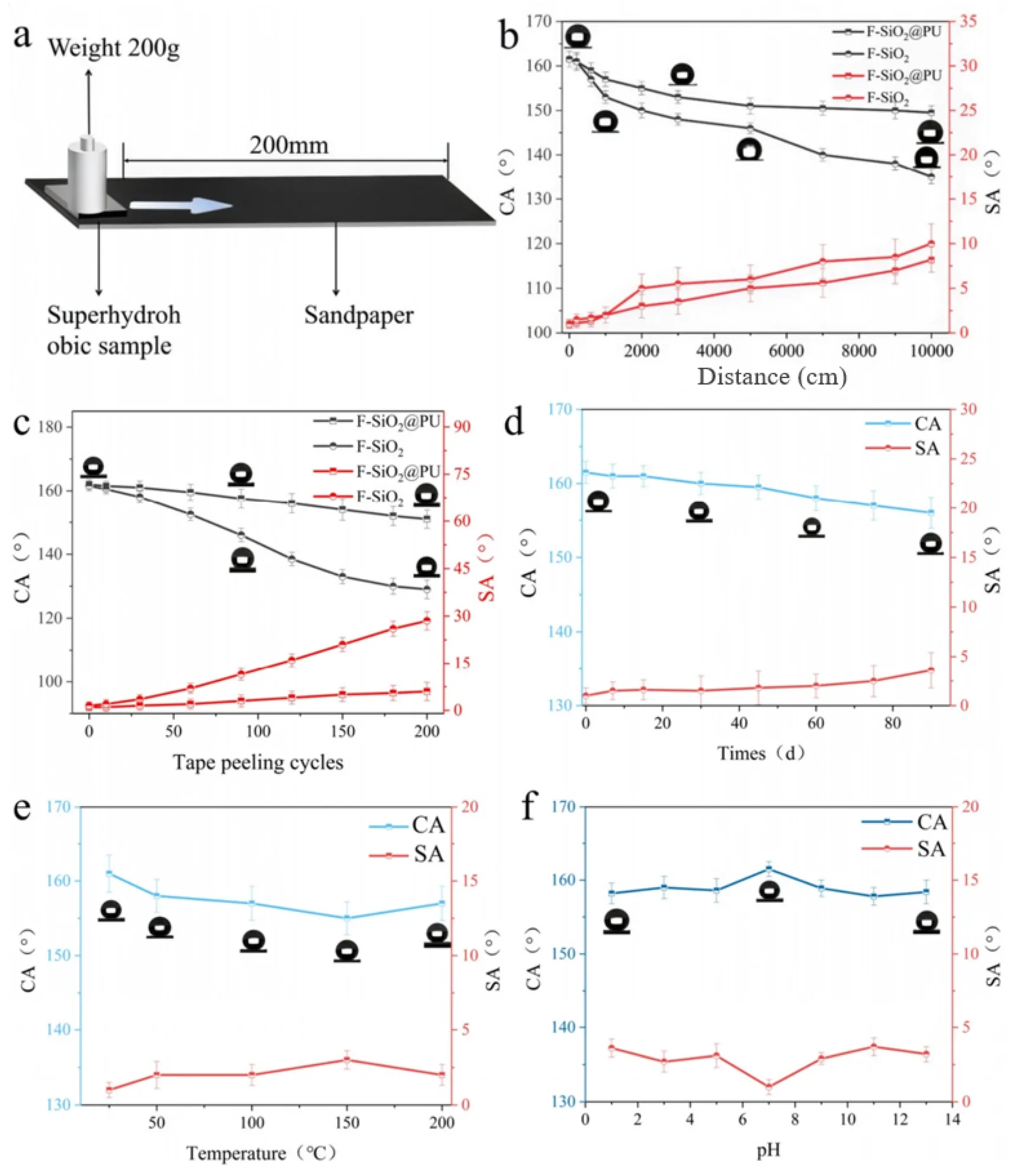
(**a**) Schematic diagram of friction and wear; (**b**) friction and wear test; (**c**) tape-peeling test; (**d**) indoor exposure test; (**e**) thermal stability test; (**f**) chemical stability test. Error bars in (**b**–**f**) represent the standard deviation of three independent measurements.

Mechanical durability is one of the most critical requirements for the practical application of superhydrophobic coatings. To evaluate the wear resistance of the F-SiO_2_@PU composite coating, abrasion tests were performed using 2000-grit SiC sandpaper under a constant load of 200 g (Figure 6a). The variations in water contact angle (CA) and sliding angle (SA) with abrasion distance are presented in Figure 6b.

For both coatings, the CA gradually decreased, while the SA increased with increasing abrasion distance, indicating progressive damage to the surface micro/nanostructures and partial removal of the low-surface-energy fluorinated layer. Such deterioration inevitably weakens the air-trapping capability of the hierarchical structure and increases the actual liquid–solid contact area. However, the F-SiO_2_@PU composite coating exhibited significantly superior abrasion resistance to the F-SiO_2_ coating. Even after an abrasion distance of 10,000 cm, the F-SiO_2_@PU coating maintained a CA of 150 ± 2° and an SA of 8 ± 1.7° (mean ± standard deviation, n = 3). In contrast, the F-SiO_2_ coating experienced a substantial loss of superhydrophobicity, with the CA decreasing to approximately 135 ± 2° and the SA exceeding 10°. The error bars in Figure 6b represent the standard deviations of three independent measurements. The same trend was observed in three independent abrasion tests, confirming the reproducibility of the wear results.

The adhesion stability of the coating was further evaluated by repeated tape-peeling tests, and the evolution of CA and SA with cycling number is shown in Figure 6c. A 200-cycle test was employed as a rigorous accelerated adhesion test. At this endpoint, the control F-SiO_2_ coating had already lost superhydrophobicity (CA ≈ 129 ± 2°, SA ≈ 29 ± 1.4°), whereas the F-SiO_2_@PU coating still maintained CA = 151 ± 2° and SA = 6 ± 1.5°. For the F-SiO_2_@PU coating, the CA decreased only slightly, and the SA increased gradually in the early stage (approximately 0–50 cycles), which may be attributed to the removal of a small amount of weakly attached surface particles. With further cycling (approximately 50–150 cycles), the changes remained relatively gradual, indicating that the PU matrix and the hydroxyl-rich anodized interface effectively anchored the fluorinated SiO_2_ particles and preserved the hierarchical structure [37]. In the later stage (approximately 150–200 cycles), the F-SiO_2_@PU coating still retained its superhydrophobic character, while the F-SiO_2_ coating exhibited a transition from the Cassie–Baxter state toward a partially wetted state. In contrast, the F-SiO_2_ coating degraded markedly throughout the test, with the CA decreasing to approximately 129 ± 2° and the SA increasing to 29 ± 1.4° after 200 cycles. The overall durability was further verified by 10,000 cm sandpaper abrasion, 90-day ambient storage, thermal exposure at 50–200 °C for 2 h, and pH 1–14 chemical stability tests. Therefore, the 200-cycle tape-peeling result, together with the multi-condition durability tests, provides comprehensive evidence for the enhanced adhesion and long-term stability of the F-SiO_2_@PU coating. Moreover, the hydroxylated anodized surface provides a rough and polar interface favorable for interaction with the PU matrix, contributing to the overall structural stability of the composite coating.

The superior anti-peeling performance further demonstrates the enhanced structural stability of the composite coating. The PU matrix improves the structural integrity of the coating, while the polymer-assisted fixation of fluorinated nanoparticles suppresses the removal of the functional surface layer during repeated peeling.

Long-term storage stability is another important indicator for evaluating the practical applicability of superhydrophobic coatings. Therefore, the wettability evolution of the F-SiO_2_@PU coating was monitored over 90 days under ambient laboratory conditions. As shown in Figure 6d, the CA decreased only slightly from 161.5 ± 2° to 156 ± 2°, while the SA remained below 4°. Such minimal variation indicates that neither significant structural collapse nor fluorosilane desorption occurred during long-term storage. The retained superhydrophobicity further demonstrates the excellent environmental durability and long-term stability of the coating.

Thermal stability was investigated by exposing the coating to temperatures ranging from 50 to 200 °C for 2 h. As shown in Figure 6e, the coating maintained a CA above 155° and an SA below 3° throughout the entire temperature range. The negligible change in wettability indicates that the fluorinated SiO_2_ nanostructure and PU matrix retained their structural stability under thermal exposure. This behavior is consistent with the excellent thermal stability revealed by the TGA results, further indicating the potential of the coating for applications under elevated-temperature conditions.

The chemical stability of the coating was further evaluated over a wide pH range from 1 to 14. As shown in Figure 6f, the CA remained within 157–161.5°, while the SA exhibited only minor fluctuations. Even under strongly acidic or alkaline conditions, the coating retained its superhydrophobicity, indicating excellent resistance to chemical degradation. The robust micro/nanostructure and chemically stable fluorinated surface layer contributed to maintaining the Cassie–Baxter wetting state, thereby preventing complete wetting of the surface.

Overall, the F-SiO_2_@PU composite coating exhibits excellent resistance to abrasion, peeling, thermal exposure, prolonged storage, and chemical attack [38]. These durability characteristics can be attributed to the synergistic effects of the hard-anodized interface, the flexible PU binding network, and the fluorinated hierarchical rough structure. The combination of strong interfacial adhesion, efficient stress dissipation, and stable surface roughness enables the coating to maintain its superhydrophobicity under various harsh service conditions, thereby providing favorable conditions for long-term corrosion protection.

### 3.4. Corrosion Protection Performance

**Figure 7:**
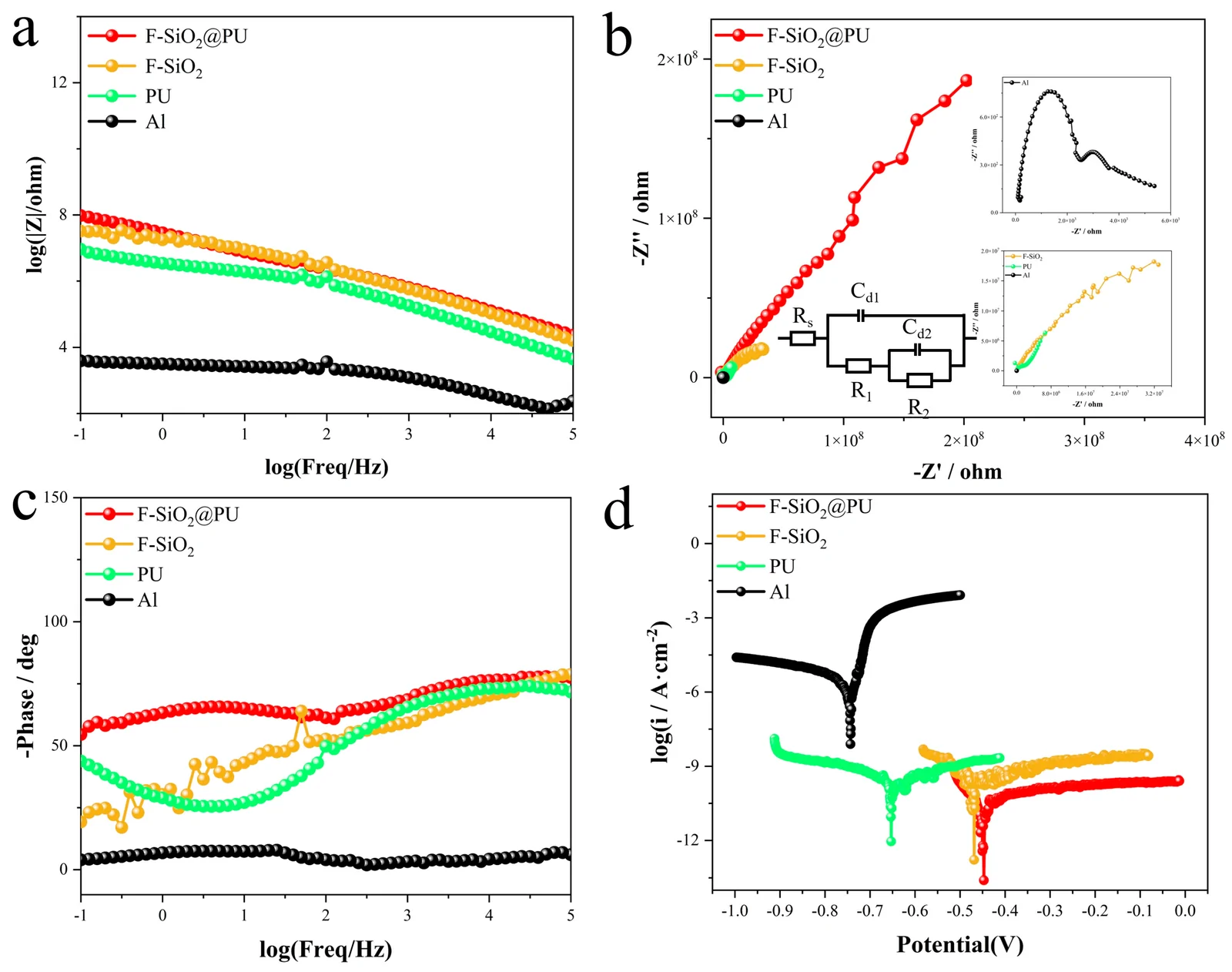
Impedance plots (**a**), Nyquist curves (**b**), Bode plots (**c**), and polarization curves (**d**) of different coatings on aluminum alloy.

The corrosion protection performance of bare aluminum alloy (Al), PU coating, F-SiO_2_ coating, and F-SiO_2_@PU composite coating was systematically evaluated in 3.5 wt% NaCl solution using electrochemical impedance spectroscopy (EIS) and potentiodynamic polarization tests. The corresponding Nyquist and Bode plots, together with the potentiodynamic polarization curves, are presented in Figure 7.

The impedance modulus is widely recognized as an important indicator for evaluating the barrier properties of protective coatings. As shown in Figure 7a, the F-SiO_2_@PU composite coating exhibited the highest impedance modulus over the entire frequency range, indicating enhanced resistance to electrolyte penetration. In particular, the low-frequency impedance modulus (log|Z|_0.1Hz_) reached 8.0, which was significantly higher than the corresponding values for the F-SiO_2_ coating (7.7), PU coating (6.9), and bare aluminum alloy (3.6). Since low-frequency impedance is closely associated with the ability of the coating to inhibit electrolyte penetration and interfacial electrochemical reactions, the high low-frequency impedance modulus further confirms the excellent barrier performance of the composite coating.

Further evidence is provided by the Nyquist plots shown in Figure 7b. The F-SiO_2_@PU coating displayed the largest capacitive semicircle radius among all samples, indicating a substantially increased charge-transfer resistance and a lower corrosion reaction rate at the coating–substrate interface. In contrast, the bare aluminum alloy exhibited only a small capacitive loop, suggesting limited corrosion resistance in the presence of aggressive chloride ions. The enlarged semicircle observed for the composite coating further demonstrates the effective suppression of interfacial electrochemical reactions.

The Bode phase-angle plots shown in Figure 7c provide additional insight into the protective behavior of the coatings. The F-SiO_2_@PU coating maintained phase angles between approximately 55° and 80° over a broad frequency range, with a maximum approaching 80°. Such behavior is characteristic of a highly capacitive response and indicates excellent barrier performance. By comparison, the PU and F-SiO_2_ coatings exhibited lower phase angles, particularly in the medium- and low-frequency ranges, indicating weaker barrier properties against electrolyte penetration. The bare aluminum alloy maintained phase angles below 10° throughout the frequency range, indicating its limited corrosion resistance in the NaCl solution.

Potentiodynamic polarization measurements were conducted to quantitatively evaluate the corrosion behavior of the different samples. As shown in Figure 7d, bare aluminum alloy exhibited a corrosion potential (E*_corr_*) of −0.76 V and a corrosion current density (I*_corr_*) of 1.87 × 10^−6^ A cm^−2^, indicating a strong tendency toward corrosion. The application of the PU coating shifted the corrosion potential positively to −0.66 V. It reduced the corrosion current density to 2.36 × 10^−9^ A cm^−2^, demonstrating the protective effect of the dense polymer barrier. Similarly, the F-SiO_2_ coating exhibited an E*_corr_* value of −0.47 V and an I*_corr_* value of 6.35 × 10^−9^ A cm^−2^, indicating substantially suppressed corrosion activity compared with bare aluminum alloy [39].

Among all samples, the F-SiO_2_@PU composite coating exhibited the most positive corrosion potential (−0.44 V) and the lowest corrosion current density (5.36 × 10^−11^ A cm^−2^). Compared with bare aluminum alloy, the corrosion current density decreased by approximately five orders of magnitude, confirming the exceptional corrosion protection capability of the composite coating. Such a dramatic reduction in corrosion activity demonstrates that the coating effectively isolates the metallic substrate from the corrosive environment.

### 3.5. Long-Term Corrosion Resistance in Acidic, Alkaline, and Saline Media

To evaluate the long-term corrosion protection capability of the F-SiO_2_@PU composite coating under harsh service conditions, immersion tests were conducted in 0.1 M HCl, 1 M NaOH, and 3.5 wt.% NaCl solutions for 7 and 14 days. The evolution of electrochemical impedance and potentiodynamic polarization behavior was systematically analyzed to reveal the degradation mechanisms of the coating in different corrosive environments.

#### 3.5.1. Corrosion Behavior in Acidic Environment

**Figure 8:**
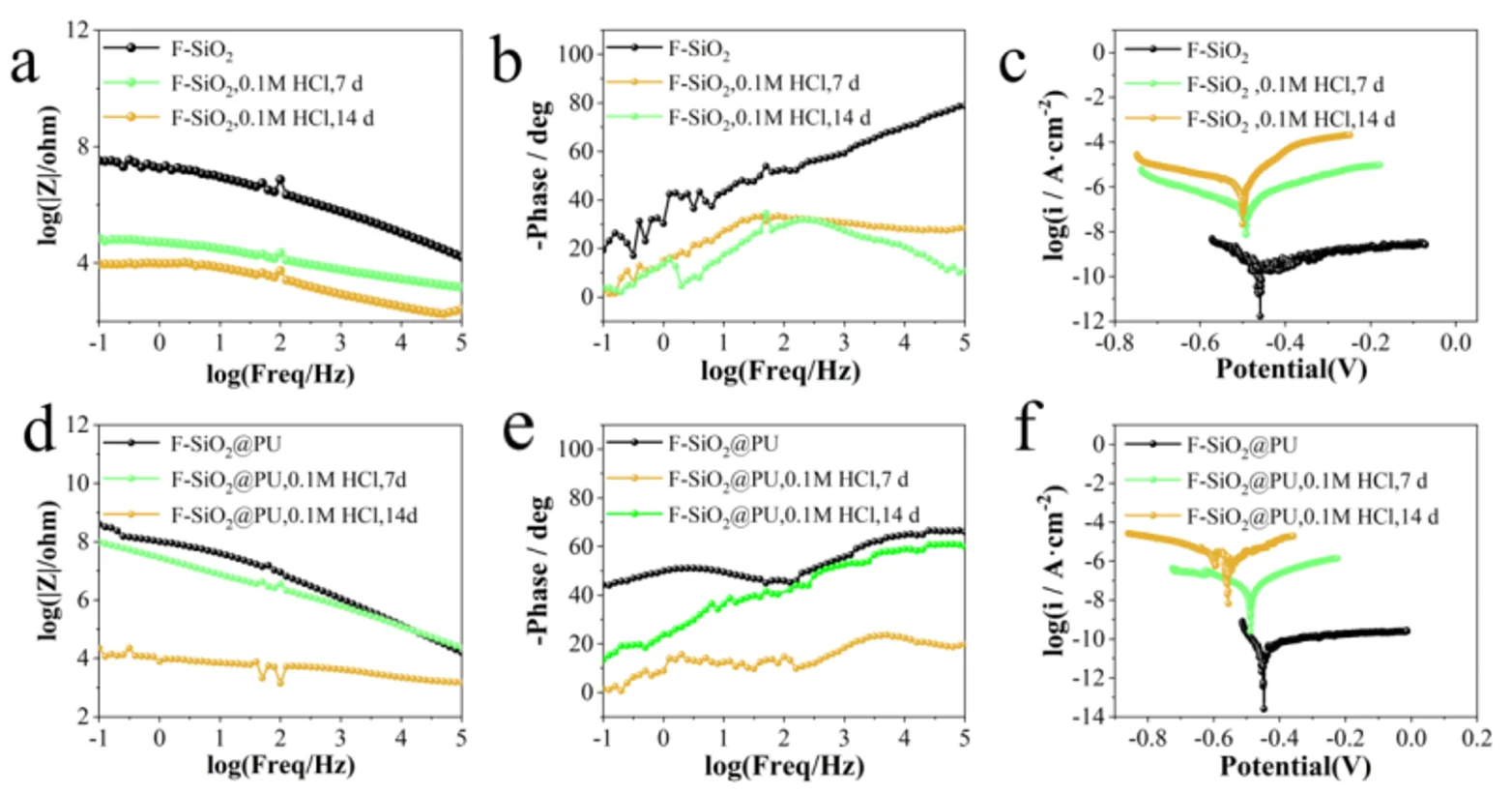
Impedance plot (**a**), phase angle plot (**b**), and polarization curve plot (**c**) of the F-SiO_2_ coating in the 0.1 M HCl immersion test; impedance plot (**d**), phase angle plot (**e**), and polarization curve plot (**f**) of the F-SiO_2_@PU coating.

The electrochemical performance of the F-SiO_2_@PU coating after immersion in 0.1 M HCl solution is shown in Figure 8. As the immersion time increased, a gradual decrease in impedance modulus and a slight increase in corrosion current density were observed, indicating the progressive penetration of corrosive species through the coating. Nevertheless, even after 14 days of exposure, the coating maintained a low-frequency impedance modulus above 10^6^· Ω·cm^2^ and a corrosion current density significantly lower than that of bare aluminum alloy.

The degradation process in acidic media is primarily associated with the penetration of aggressive H^+^ and Cl^−^ ions through defects and pores in the coating. As immersion proceeds, these corrosive species can gradually reach the coating–substrate interface, leading to localized dissolution of the surface oxide layer and subsequent corrosion of the aluminum substrate. In addition, prolonged acidic exposure may promote the hydrolysis of susceptible chemical bonds within the coating and at the interface, thereby compromising the structural integrity and barrier properties of the coating.

Despite these challenges, the corrosion resistance of the composite coating remained at a relatively high level throughout the immersion period. The dense PU matrix effectively slowed proton transport, while the fluorinated superhydrophobic surface reduced electrolyte adhesion and retention on the coating surface. Furthermore, the trapped air layer within the hierarchical roughness reduced the effective contact area between the acidic solution and the coating, thereby delaying electrolyte penetration and suppressing interfacial corrosion reactions [40].

#### 3.5.2. Corrosion Behavior in Alkaline Environment

**Figure 9:**
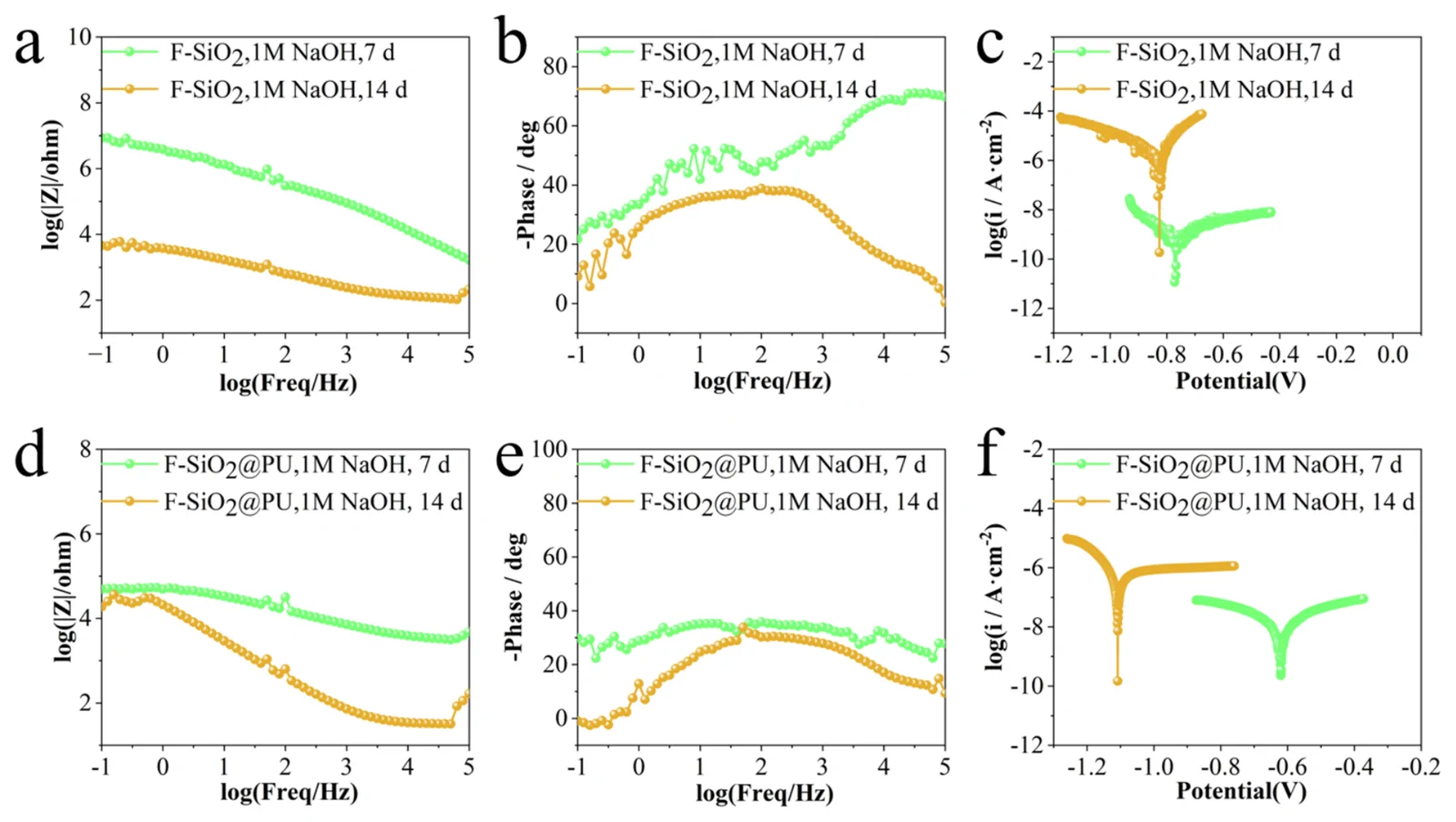
The impedance plot (**a**), phase angle plot (**b**), and polarization curve plot (**c**) of the F-SiO_2_ coating after immersion in a 1 M NaOH solution, as well as the impedance plot (**d**), phase angle plot (**e**), and polarization curve plot (**f**) of the F-SiO_2_@PU coating.

Figure 9 presents the electrochemical behavior of the coating after immersion in 1 M NaOH solution. Compared with acidic conditions, a more pronounced decrease in the impedance modulus was observed as immersion time increased, indicating that the alkaline environment caused more severe degradation of the protective performance of the coating.

The degradation mechanism under alkaline conditions differs substantially from that in acidic environments. Hydroxide ions (OH^−^) can attack siloxane bonds within the fluorinated silica network, gradually weakening the integrity of the micro/nanostructure. Simultaneously, prolonged exposure to strong alkali may induce partial hydrolysis of urethane linkages within the PU matrix, resulting in localized softening of the polymer barrier. Such processes increase the probability of electrolyte transport through the coating.

Despite the aggressive alkaline environment, the F-SiO_2_@PU coating still maintained relatively high impedance and low corrosion current densities after 14 days of immersion. This behavior indicates that the synergistic protection provided by the composite coating remained effective during prolonged alkaline exposure. The fluorinated surface continued to provide substantial liquid repellency, while the PU layer retained sufficient structural integrity to limit electrolyte penetration and ionic transport. Moreover, the anodized layer beneath the coating acted as an additional protective barrier, further delaying substrate degradation.

#### 3.5.3. Corrosion Behavior in Saline Environment

**Figure 10:**
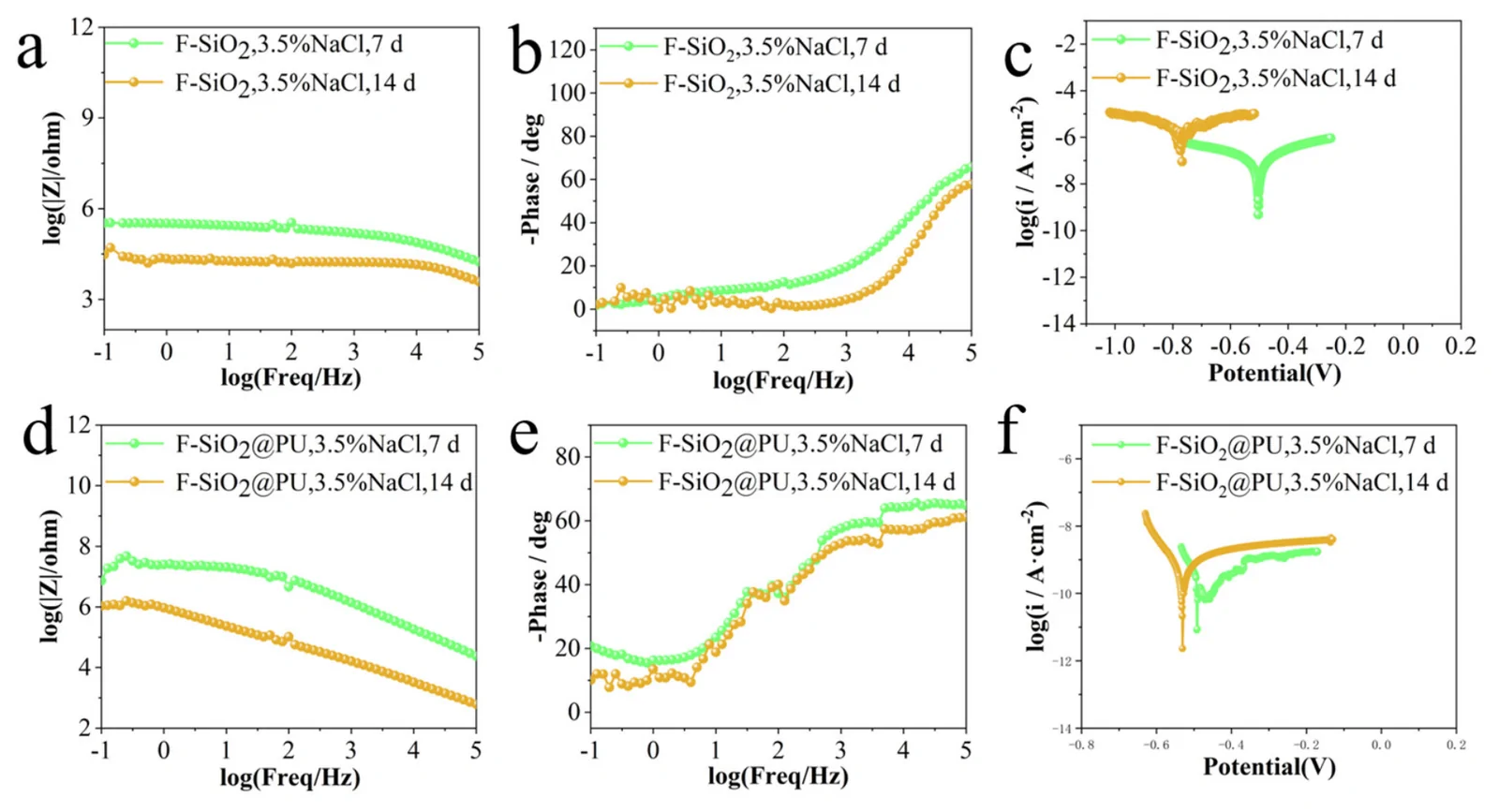
Impedance plot (**a**), phase angle plot (**b**), and polarization curve plot (**c**) of the F-SiO_2_ coating after immersion in a 3.5 wt.% NaCl solution; the impedance plot (**d**), phase angle plot (**e**), and polarization curve plot (**f**) of the F-SiO_2_@PU coating.

The corrosion resistance of the coating in a 3.5 wt.% NaCl solution is shown in Figure 10. Compared with acidic and alkaline environments, the coating exhibited the highest electrochemical stability in saline solution. After 7 and 14 days of immersion, only slight variations in the impedance modulus and corrosion current density were detected.

The primary corrosive species in saline environments are chloride ions (Cl^−^), which are well known for their ability to penetrate passive films and initiate localized corrosion on aluminum alloys. For conventional coatings, the gradual diffusion of chloride ions often leads to interfacial delamination and pitting corrosion. In contrast, the F-SiO_2_@PU coating effectively restricted chloride-ion transport through the synergistic action of multiple protective barriers.

The superhydrophobic top layer substantially reduced the effective contact area between the electrolyte and the coating surface [41]. Meanwhile, the trapped air layer within the hierarchical micro/nanostructure acted as an additional barrier to electrolyte penetration, effectively prolonging the transport pathways of chloride ions. Even when limited amounts of electrolyte penetrated the outer layer, the dense PU matrix effectively restricted further migration toward the substrate. Consequently, the corrosion activity at the coating/substrate interface remained at a relatively low level throughout the immersion period.

### 3.6. Wear-Resistant Superhydrophobic Corrosion Protection Mechanism

**Figure 11:**
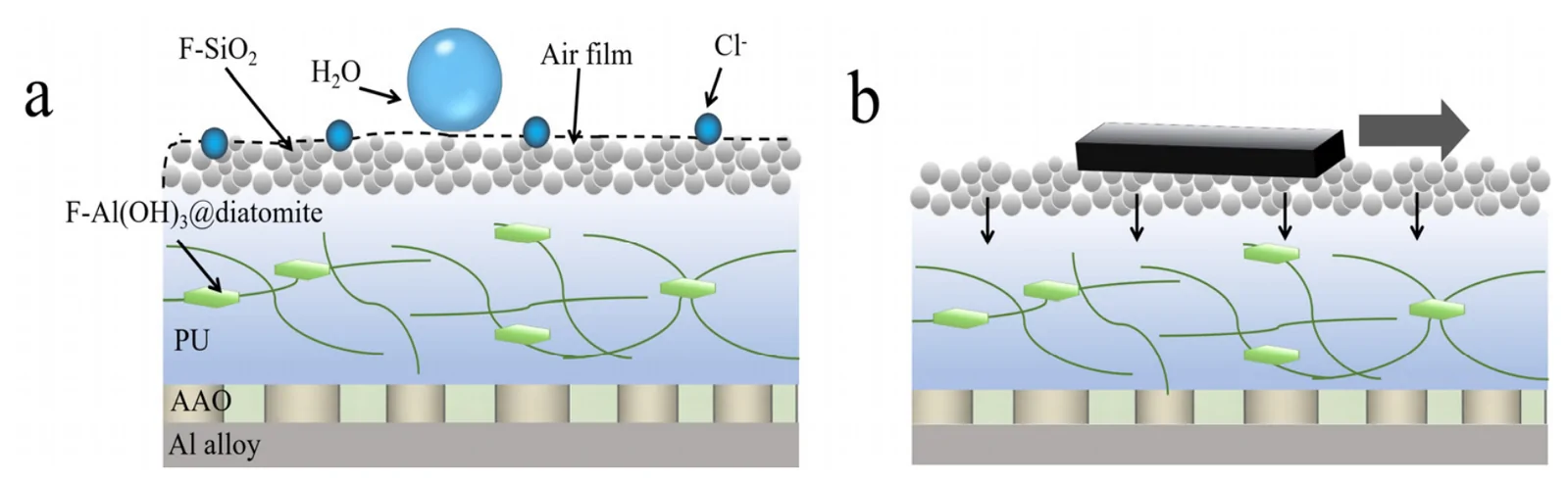
(**a**) Mechanism diagram of the superhydrophobic and corrosion-resistant properties of the F-SiO_2_@PU coating; (**b**) mechanism diagram of the wear-resistant properties of the F-SiO_2_@PU coating.

The superior performance of the F-SiO_2_@PU coating arises from the synergistic integration of a hard-anodized interfacial layer, an F-Al (OH)_3_@diatomite-reinforced PU interlayer, and a fluorinated F-SiO_2_ top layer (Figure 11). The anodized surface provides abundant anchoring sites, while the embedded F-Al (OH)_3_@diatomite particles act as micron-scale reinforcing fillers within the PU matrix [42,43]. Meanwhile, the continuous PU network contributes to particle fixation, stress dissipation, and barrier protection. The fluorinated F-SiO_2_ top layer provides nanoscale roughness and low surface energy, enabling a stable Cassie–Baxter state and effective air trapping. Among these factors, the interfacial anchoring provided by the hard-anodized layer is likely the most critical, because it determines the coating–substrate adhesion and structural integrity, thereby enabling the other protective effects to function under mechanical and corrosive conditions. Therefore, the combined effects of interfacial anchoring, PU-based structural reinforcement, and the superhydrophobic air barrier contribute to the enhanced mechanical durability and corrosion protection of the coating.

## 4. Conclusions

A wear-resistant superhydrophobic F-SiO_2_@PU composite coating was successfully fabricated on hard-anodized aluminum alloy via a facile spray-coating process. Benefiting from the synergistic integration of the hard-anodized interface, particle-reinforced PU interlayer, and fluorinated hierarchical top layer, the coating exhibited excellent superhydrophobicity, mechanical durability, and corrosion resistance. At an FDTS dosage of 0.3 mL, uniformly distributed fluorinated SiO_2_ particles generated a well-developed rough structure, resulting in a water contact angle of 161.5 ± 2° and an ultralow sliding angle of less than 2° (approximately 1°). The incorporation of the PU layer significantly enhanced interfacial adhesion and structural integrity, enabling the coating to retain a contact angle of approximately 150° after 10,000 cm abrasion and 200 tape-peeling cycles. Electrochemical measurements demonstrated outstanding corrosion protection performance, with a low-frequency impedance modulus of log|Z|_0.1Hz_ = 8.0 and an ultralow corrosion current density of 5.36 × 10^−11^ A cm^−2^ in 3.5 wt.% NaCl solution, representing a reduction of approximately five orders of magnitude compared with bare aluminum alloy. Furthermore, the coating maintained high corrosion resistance in acidic, alkaline, and saline environments. The enhanced performance can be attributed to the strong interfacial bonding provided by the anodized layer, the dense barrier effect of the PU matrix, and the stable air-trapping capability of the fluorinated SiO_2_ hierarchical structure, which collectively inhibit the ingress of corrosive species and improve resistance to mechanical damage. These findings demonstrate that the integrated multilayer design provides an effective strategy for simultaneously enhancing the mechanical durability and long-term corrosion protection of superhydrophobic coatings on aluminum alloys.

## Data Availability

The original contributions presented in this study are included in the article/Supplementary Materials. Further inquiries can be directed to the corresponding authors.

## Supplementary Materials

The following supporting information can be downloaded at: https://www.mdpi.com/article/10.3390/ma19194155/s1, Figure S1. SEM images of F-SiO_2_ nanoparticles prepared with different FDTS dosages: (a) 0.1 mL, (b) 0.2 mL, (c) 0.3 mL, (d) 0.4 mL, and (e) 0.5 mL; (f) corresponding CA and SA. Figure S2. SEM images of the F-SiO_2_ superhydrophobic coating (a–c) and the F-SiO_2_@PU composite superhydrophobic coating (d–f).
