# Supplementary material for: Interfacial Regulation of Hard-Anodized Aluminum Alloy for Fabrication of a Wear-Resistant Superhydrophobic F-SiO2@PU Composite Coating with Enhanced Long-Term Corrosion Protection
Source: Materials (Basel). 2026 Sep 29;19(19):4155. doi: 10.3390/ma19194155 (PMC13666378; doi:10.3390/ma19194155)
Supplement: Supplementary file 1 [file materials-19-04155-s001.zip › materials-4569078-supplementary.pdf]

Interfacial Regulation of Hard-Anodized Aluminum Alloy for Fabrication of a Wear-Resistant Superhydrophobic F-SiO_2@PU Composite Coating with Enhanced Long-Term Corrosion Protection

Hong-Bo Wang [1,2,*], Miao Yu [1], Chong Ren [1], Muhaiminul Islam Shakil [1], Chen Chen [1], Liang Zhou [1,*], Da-Yan Ma [3,*] and Wei Wang [4]

[1] School of Materials Science and Engineering, Chang'an University, Xi'an 710064, China
[2] Shaanxi Key Laboratory of Carbon Dioxide Sequestration and Enhanced Oil Recovery, Xi'an 710075, China
[3] State Key Laboratory for Mechanical Behavior of Materials, Xi'an Jiaotong University, Xi'an 710049, China
[4] State Key Laboratory of Clean and Efficient Turbomachinery Power Equipment, Dongfang Turbine Co., Ltd., Deyang 618000, China
* Correspondence: wanghb@chd.edu.cn (H.-B.W.); zhouliang@chd.edu.cn (L.Z.); madayan@mail.xjtu.edu.cn (D.-Y.M.)

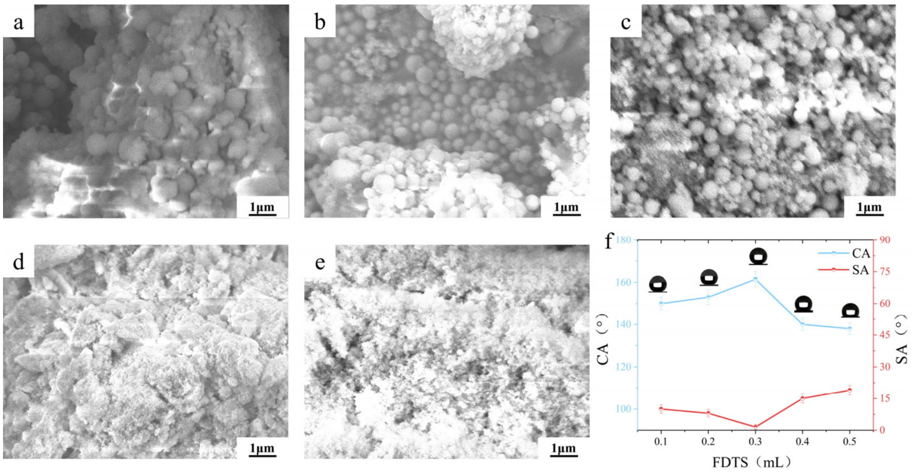

Figure. S1. SEM images of F-SiO_2 nanoparticles prepared with different FDTS dosages: (a) 0.1 mL, (b) 0.2 mL, (c) 0.3 mL, (d) 0.4 mL, and (e) 0.5 mL; (f) corresponding CA and SA.

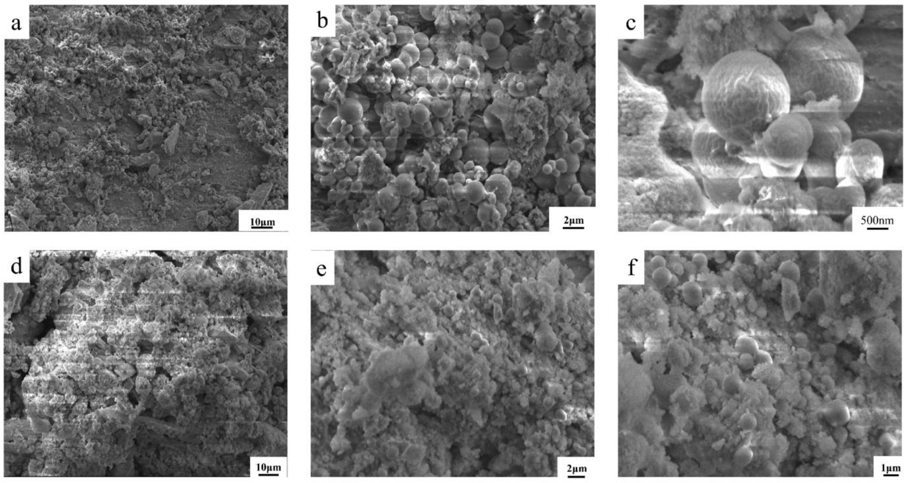

Figure. S2. SEM images of the F-SiO_2 superhydrophobic coating (a-c) and the F-SiO_2@PU composite superhydrophobic coating (d-f).
